# Participatory Health Impact Assessment for Health and Well‐Being Policy at Local Level in Thailand

**DOI:** 10.1002/hpja.70073

**Published:** 2025-07-13

**Authors:** Nattaya Promthong, Suwicha Thaweesook, Phen Sukmak, Sirima mongkolsomlit, Patchana Hengboriboonpong Jaidee, Weerasak Putthasri

**Affiliations:** ^1^ National Health Commission Office Nonthaburi Thailand; ^2^ Public Policy Institute Prince of Songkhla University Songkhla Thailand; ^3^ Faculty of Public Health Thammasat University Pathum Thani Thailand; ^4^ Faculty of Public Health Burapha University Chonburi Thailand

## Abstract

**Issue Addressed:**

Achieving the Sustainable Development Goals (SDGs) related to health and well‐being requires diverse stakeholders' engagement. According to Thailand's constitution, the decentralisation of health promotion to Local Administrative Organizations (LAOs) has been increasingly implemented. The lessons learned from experiences in implementing well‐being tools and engaging stakeholders in diverse local and cultural contexts offer practical benefits. This article describes the development of participatory policy using health impact assessment (HIA) in selected LAOs across Thailand, alongside bottom‐up policy formulation, to support sustainable social well‐being and community empowerment.

**Methods:**

A qualitative research approach was applied to examine HIA implementation in 12 purposively selected LAOs across Thailand. Data collection included document reviews, participatory observations of HIA meetings/activities and 156 in‐depth interviews with key informants (KIs) who were actively involved in the HIA process. The analysis specifically examined the roles of community and stakeholder engagement, the implementation processes and the resulting outcomes.

**Results:**

The 12 LAOs selected a diverse range of challenges for HIA applications, including waste management, water pollution, elderly care, occupational health and community tourism. Community consultation with government personnel, academics and community members was conducted as the preparation phase to identify priority issues. Most participants emphasised that meaningful and inclusive participation through the HIA process was crucial for creating ownership and ensuring compliance with agreements. The influencing step of HIA was significant in securing commitment and continued support from local governments. Participants also mentioned increased motivation, active citizenship, community empowerment and collective leadership development.

**Conclusions:**

The findings indicate that the HIA is a social tool effectively implemented in the community. Various issues and challenges of health and well‐being are manifest in deployment.

**So What?:**

Inclusive participation through the policy development process at the local level is clarity to support sustainable social well‐being and community empowerment.

## 1 Introduction

The World Health Organisation (WHO) defines the health impact assessment (HIA) as “a practical approach used to assess the potential health effects of a policy, programme or project on a population, particularly on vulnerable or disadvantaged groups.” HIA is a systematic tool that can be used by decision‐makers and stakeholders to support evidence‐informed decision‐making to maximise positive health effects and minimise negative impacts of policies, programmes or projects. HIA is conducted by multidisciplinary teams comprising representatives from decision‐making bodies, affected communities and health experts to ensure comprehensive assessment and to integrate information from both scientific literature and stakeholder consultations. This collaborative and participatory tool can be applied to national policy appraisal, local planning and other development projects. The National Health Service (NHS) regions and local authorities in the United Kingdom have already integrated HIA into their practices [1].

The Seventy‐seventh World Health Assembly [2] had endorsed the resolution to implement ‘the meaningful social participation in decision‐making processes for health. Social Participation, as defined by WHO, involves empowering people, communities and civil society through inclusive participation in decision‐making processes that affect health across the policy cycle and at all levels of the system’. This global momentum emphasises the practical of participatory and inclusive health governance toward SDGs vision. It might be said that SDGs and Health and well‐being, in particular, definitely requires dynamic stakeholder engagement and cross‐sector collaboration [3]. However, more evidence is needed to highlight practical knowledge and participatory tools that facilitate effective participation.

According to the Ottawa Charter in 1986, it emphasised that healthy public policy is a key component of health promotion. The HIA was recommended as a tool to assess the health consequences in support of those policy decisions [4]. The International Association for Impact Assessment (IAIA) mentions that HIA has evolved from being a part of environmental impact assessment and an approach for intersectoral action on the broader determinants of health to address health inequities [5]. The methods of HIA are mostly described in five steps [6], that is, (1) Screening (determine if HIA is required); (2) scoping (which impacts will be considered); (3) identification and assessment of impacts (determine the magnitude, nature, extent, potential health impacts); (4) influencing or decision‐making and recommendation (making explicit the trade‐offs and policy recommendations); (5) evaluation, monitoring and follow‐up (process of impact evaluation).

HIA is increasingly applied as a core aspect of health promotion practice, as it offers the health sector a structured, transparent method and process to work with other sectors through formulating and implementing of healthy public policy [7]. Harris‐Roxas [8] presented a diverse range of experiences and practices, for example, climate change mitigation policies at the local level in Switzerland, transport and urban planning in France, e‐waste in Sub‐Saharan Africa.

Dannenberg [9] indicated that the major impacts of HIAs were directly influencing some decisions, improving collaboration among stakeholders, raising health awareness among decision makers and giving community members a stronger voice in local decisions. The effectiveness of HIA also included the ability to change or influence decisions, building strong and enduring relationships with other sectors, and developing a clear understanding of the perspectives of partners [10].

The community participation is generally considered a core element in HIA to get value from local knowledge, adherence to democratic values and empowerment of communities [11]. This community participation improved relations between communities and local agencies, policy makers and professionals, and the empowerment of community members.

It is accepted that local authorities have been increasing their role in health promotion and well‐being in society. Pettman et al. [12], suggested that health promotion programmes for local governments should include systems for access to academic literature; processes that make it easier to use evidence; training in evidence‐informed health promotion to build organisational culture and capacity; and research‐practice partnerships and mentoring.

Local governments have the significant role to improve health and wellbeing in their catchment areas with sharing of societal goal of equitable wellbeing and proportionate actions [13]. The local governments in New South Wales and Victoria, Australia [14] were reported implementing in their role in a wide range of initiatives, for example, food access, healthy food, drink options. Partnerships were highlighted as an enabler of those policies and programs.

There is evidence of local authorities using HIA as a tool to develop local health policies. The local authorities in the North East of England [15] demonstrated that HIA could be brought into the local policy‐making and planning activities and helped them achieve a broad range of corporate health objectives. There was a study stating that HIA can be carried out for local policies in a simple, flexible and effective way [16]. It could facilitate reflection on the SDHs and the health impacts of local authority initiatives. It mentioned that genuine community participation in the HIA process was critical.

It was also evidenced influences on the use of HIA knowledge on health promotion and community planning activities in Northern Ireland and the Republic of Ireland [17] to engage the public authorities and external partners assisted implementation of the HIA findings. HIA was introduced aiming to integrate health issues on the political agenda and helping to reduce health inequalities. The HIA has been implemented in Swedish municipalities [18]. After 15 years implementation, the political context is emphasised as one of crucial factors for HIA public health practice at the local level. In local development plan, HIA was suggested to us as a tool for participation [19]. This platform meeting with a wide range of health and well‐being representatives could take place and be part of the local mandatory consultation events.

In Thailand, HIA has gained significant traction and emerged from widespread support for ‘healthy public policy’ in Thailand it was legislated in the National Health Act (2007). The community‐led HIA process (CHIA) in Thailand emphasises the importance of community learning about the impacts of planned projects and policies on community health, and therefore can be viewed as an empowerment process [20]. The CHIA has long been conducted in Thailand with methods of social norm and belief oriented [21]. It created a bottom‐up health promotion in which communities collectively make it up through developing healthy public policies, strengthening communities and creating an enable environment. It was concluded that HIA in community created a mutual learning of all sectors through practices and development and a tool for empowerment of civil society.

Since 1999, Thailand has gradually implemented the health‐care decentralisation, which meant a transfer of authority and responsibility from the central government to the local government units. It was agreed with less argument that the responsibility for the provision of primary care, including health promotion, could be transferred to local units to fit their capacity and was consistent with health‐care decentralisation in developed countries [22]. Therefore, the evidence on how the local agencies performed for sustainable social well‐being should be explored.

Ministry of Public Health, Thailand had issued the guideline of HIA for local authority since 2013 [23]. However, the outcome or effectiveness from those HIA guideline applications for local public policy development in supporting implementation on health promotion under their authorisation needed to be documented.

This study aims to: (1) analyse the operating models of HIA implementation in Thai LAOs; (2) document lessons learned from diverse local contexts and (3) identify the approach of HIA into the decision‐making and implementation in their settings.

## 2 Methods

### 2.1 Study Design

A qualitative research approach was applied to examine HIA implementation across 12 purposively selected local administrative organizations (LAOs) in Thailand that had implemented community‐led HIA (CHIA) between June 2023 and May 2024.

### 2.2 Case Selection

LAOs were selected by selective sampling to ensure diversity across:

- Geographic regions (Northern, Central, Eastern, Northeastern and Southern Thailand)
- Administrative levels (municipalities and Provincial Administrative Organizations)
- Health issues addressed through HIA

### 2.3 Data Collection

Data were collected through three complementary methods:

1. Document review: Analysis of HIA reports, meeting minutes, policy documents and project materials from each LAO.
2. Participatory observation: researchers attended HIA meetings, community consultations and policy forums, recording field notes on processes, interactions and decision‐making dynamics.
3. In‐depth interviews: Semi‐structured interviews (*n* = 156) were conducted with:
  ○ Local government administrators (*n* = 48)
  ○ Related government agencies (*n* = 12)
  ○ Academic facilitators from partner universities (*n* = 12)
  ○ Community members involved in HIA processes (*n* = 60)
  ○ Civil society networks and related stakeholders (*n* = 24)

### 2.4 Data Analysis

Content analysis was conducted, focusing on three key aspects, that is, the selected challenge issues for HIA application, the operating models used in implementing HIA, and the improvements in HIA performance and benefits for the local administrative organizations (LAOs) and communities.

## 3 Results

A total of 12 LAOs implemented the community‐led HIA. The community consultation with the evidence supports from academic institutes was initially vital for issue selection. A wide range of challenging issues were selected by 12 LAOs for HIA application includingwaste management, water pollution, elderly care, occupational health, community tourism and primary care devolution. The conditions for selecting issues included development policy direction, prioritised health problems and citizen requests or complaints regarding problematic issues in the area (Table 1).

**TABLE 1:** Selected local organisation, issue selected and academic institute.

Region	Province	Local administrative	Issue selected	Academic institute	No. of KIs
Northern	Lamphun	Sri Buaban Municipality	Community tourism	Faculty of Public Health, Chiang Mai U.	13
	Chiang Mai	2 On Tai Municipality	Local manufacture	Faculty of Public Health, Chiang Mai U.	13
	Sukhothai	3 Sri Satchanalai Municipality	Silverware industry	Faculty of Public Health, Naresuan U.	13
	Pitsanulok	4 Plak Rad Municipality	Waste management	Faculty of Public Health, Naresuan U.	13
Central	Saraburi	5 Saraburi Provincial Organization	Waste management	Faculty of Public Health, Thammasat U.	13
	Samut Sakorn	6 Samut Sakorn Provincial Organization	Waste water management	Faculty of Public Health, Thammasat U.	13
Eastern	Rayong	7 Tab Ma Municipality	Organic‐waste management	Faculty of Public Health, Burapha U.	13
	Chonburi	8 Chonburi Provincial Organization	Emergency and rapid response services	Faculty of Public Health, Burapha U.	13
Northeastern	Nakorn Ratchasima	9 Nakorn Ratchasima Provincial Organization	Older person care	Faculty of Public Health, Vongchavalitkul U.	13
	Chaiyaphum	10 Kon Sarn Municipality	Food security	Faculty of Public Health, Khonkaen U.	13
Southern	Yala	11 Yala Municipality	Food security	Public Policy Institute, Prince of Songkhla U.	13
	Phuket	12 Phuket Provincial Organization	Primary care devolution	Public Policy Institute, Prince of Songkhla U.	13

### 3.1 HIA Operating Models

The operating model of HIA in selected LAOs consisted of three phases as follows.

Firstly, the preparation phase involves discussing with local administrators, assigning the key actors or main workers, appointing the HIA working group, providing information and knowledge about the HIA for capacity building, setting mutual goals and making operational plans.

Secondly, the assessment phase of the HIA process consisted of five steps, that is, (1) screening, (2) scoping, (3) assessing/appraisal (this process comprised of sub‐processes including data collections and analysis, policy recommendations development and report preparation), (4) reviewing the HIA report by the relevant agency and the public to discuss the health impacts and policy recommendations to provide suggestions and additional information for the completeness of the HIA report and (5) influencing.

Thirdly, the implementation phase concentrated on the three step of HIA: driving, monitoring and evaluation.

Since the HIA of selected LAOs have just been implemented, the outcome or impact from those HIAs could not be observed yet. Initially, the short‐term effectiveness and the promising policy advocacy approaches could be determined, Table 2.

**TABLE 2:** Effectiveness and policy advocacy from HIA in selected local organisations.

Local administrative	Effectiveness and policy advocacy approaches
Sri Buaban Municipality	Established a local tourism plan and map Organised community tourism event LAO adopted HIA recommendation, then tourist information and facilities established, integrated into municipality's plan
2 On Tai Municipality	Organised a centre of local products compilation Results and recommendation from HIA were presented in the municipality meeting
3 Sri Satchanalai Municipality	Changed material‐used (asbestos free) in silver welting process Policy recommendation was adopted by related agencies and private entrepreneur
4 Plak Rad Municipality	Adopted participatory public policy on waste management and the recommendation was integrated into municipality plan
5 Saraburi Provincial Organization	Provincial Administrative Organization (PAO) high position officer were aware and committed to move the agenda through Provincial Governor for all LAOs in the province
6 Samut Sakorn Provincial Organization	PAO used the recommendation to discuss and line up with related agencies for synergic work
7 Tab Ma Municipality	Chair of the working group was the municipality board to push and scaling up the waste management agenda align with the circular economy
8 Chonburi Provincial Organization	Recommendation for setting emergency preparedness and response, and human capacity in particular
9 Nakorn Ratchasima Provincial Organization	Having measures and municipal law for chemical‐contaminated foods in marketplace Promoted school‐lunch program from safety agriculture products
10 Kon Sarn Municipality	Established integrated information system Recommendation was integrated to project of care giver training
11 Yala Municipality	Recommendation was adopted funded by municipality and community health fund
12 Phuket Provincial Organization	Policy recommendation was applied for HR management, health promoting hospital plan and service model

In the influencing step of HIA, policy recommendations and various policy options were presented to local decision‐making bodies with active community engagement. Several explicit policy changes were observed, for example, the adoption of innovative friendly‐material replace conventional asbestos in Sri Satchanalai, having municipality law on chemical‐contaminated food in Nakorn Ratchasima. All participating LAOs emphasised the benefit of HIA in using more evidence to local health policy development. It also served as an effective learning platform, fostering collaboration among policymakers, communities and academic institutions.

The entire HIA process emphasised fostering a collaborative learning environment among the involved operational mechanisms, working together to gather information and develop the policy recommendations or solutions that will jointly drive action toward development or solving problems in the area. Most participants mentioned that the meaningful and inclusive participation through the HIA process was crucial to make sense of belonging and comply with their agreement. The ‘HIA influencing’ was significant for policy commitment and continuing support from LAOs and key actors. Participants also mentioned about feeling motivated, active citizen, community empower and strengthened the networks to create collective leadership as well as developed mutual ownerships for the policies driving toward sustainable future.

### 3.2 Pattern of Learning and Capacity Development

The mode of HIA performance improvements and benefits for the LAOs and communities could be classified into three types, as follows:

#### 3.2.1 Type 1: Targeted Performance Improvement

Learning for continually improving performances focused on certain target group's performances through workshops that were designed for the identified target group. These included HIA concepts and practices by learning from case studies with the HIA mentoring academic centre.

#### 3.2.2 Type 2: Learning‐by‐Doing

Active learning how to operate and integrate from concepts into practice and action in respective local contexts.

#### 3.2.3 Type 3: Collective Empowerment Through Knowledge Exchange

Learning how mutual and collective empowerment was formed by practice experience as a ‘learning platform’ by presenting and disseminating findings from each process. Node and networks were also formed providing supports or suggestions to each other, as well as, mentoring from the academic centre. Importantly, there was the regional HIA academic centre or hub to supervise and technical support for those local implementation and network.

From key informant interviews, several key success factors and supportive environmental elements were identified, including the selection of issues, local commitment, academic engagement and intersectoral collaboration. Alignment of selected issues relevant to local or national policy direction was crucial at the beginning. Public participation awareness, concern and commitment of those local authorities ensured the agenda to be discussed in the local policy development. Academic institutes were truly engaged and supported the HIA process through providing data, information and technical capacity. Collective leadership and the establishment of an intersectoral collaboration platform in practice were also vital for effective implementation. However, a few findings on engaging the private sector and youth, dealing with the political conflict issue, as well as having various capabilities of the local agencies in terms of budget and human resources was explicitly clear.

## 4 Discussion and Interpretation

This study highlights several critical elements that contributed to the successful implementation of community‐led HIA across selected LAOs in Thailand. The findings emphasise the importance of aligning selected issues with local and national policy directions, which helped ensure that the HIA process remained relevant and that outcomes would be supported by local authorities. This alignment was crucial in fostering local commitment and ensuring that HIA outputs were integrated into local policy development.

Our findings demonstrated the operating model of HIA in 12 selective in Thailand LAOs. The HIA was clearly applied to solve the local issue challenges on health promotion and well‐being including waste management, waste‐water, elderly care, occupational health, community tourism and primary care devolution. It obviously revealed the local practice experience of well‐being tools and ways of stakeholders' working in different local contexts and cultural diversity through this participatory policy process. It was supported and relevant to other studies [19, 21, 22]; our selected local authorities were also brought forth the HIA recommendations into the decision‐making and implementation in their settings.

Public participation emerged as a key success factor, with meaningful engagement from communities and local authorities throughout the HIA process. This involvement helped create a sense of ownership over the selected issues, which in turn facilitated compliance with policy recommendations and actions. The findings support existing literature, such as Dannenberg et al. [10], which suggest that community participation strengthens relationships between stakeholders and encourages sustained commitment to health‐related decisions.

Academia's role in the HIA process was equally significant. Local academic institutions provided technical support, data and evidence, enabling local governments to make informed decisions. This collaboration not only strengthened the HIA process but also created a learning platform where communities, policymakers and academic experts could collectively contribute to the development of effective health policies. This reflects the broader body of work, including that of Harris‐Roxas [8], which demonstrates the value of evidence‐based approaches to policy formulation and the role of academia in supporting cross‐sector collaboration.

The HIA processes created the collaborative learning process among related operating mechanisms to gather information and develop policy recommendations or solutions. Good practice of meaningful and inclusive participation was vital to make sense of belonging and comply with their decision. The process of HIA influencing ran through effective policy commitment. The local HIA manifested the benefits through a learning for improving performance workshop, an active learning of how to operate and integrate concepts into practice and action in respective local contexts and learning how mutual and collective empowerment was formed by practice experience by presenting and disseminating findings from each process. Furthermore, the key success factors encompassed selected issue relevancy, local commitment, academia engagement and intersectoral practice. However, a few findings on engaging the private sector and youth, dealing with the political conflict issue, as well as having various capabilities of the local agencies in terms of budget and human resources were explicitly clear.

One of the most notable successes of the HIA implementation was the development of collective leadership and the establishment of intersectoral collaboration platforms. These platforms allowed stakeholders from various sectors to work together toward common health and well‐being goals. However, this study also uncovered several challenges, such as the limited involvement of the private sector and youth, difficulties arising from political conflicts, and disparities in local agencies' capacities, particularly concerning budgets and human resources. These findings are consistent with previous research, which often highlights the complexities of intersectoral collaboration and the challenges of achieving equitable resource allocation in decentralised governance structures [15].

The community HIA demonstrated as a health promotion platform according to the Ottawa Charter [24] of health promotion action and prerequisites for health in various issues, including environment, food, income, equity and so forth. Means of health promotion action could be implemented through HIA process, that is, building health public policy, creating supportive environment and strengthening community action.

In sum, there was an opportunity for local governments to apply HIA for achieving effective health promotion and well‐being. The knowledge obtained from our study will be beneficial for the LAOs across the country as the guidelines for HIA application as well as the learning model for academic institutes/scholars/researchers' roles to support HIA activities at the local level and work with multi‐sectoral networks for HIA and other public activities. Moreover, it is also evidence to develop a more proper supporting mechanism for HIA application in LAOs and other networks in the future.

## 5 Conclusion

The research findings indicated that the HIA is practical as a social tool effectively implemented in the community for local authority policies. Various issues and challenges of health and well‐being are manifest in deployment. It is clear that inclusive participation through the policy development process at the local level can support sustainable social well‐being achievement and community empowerment.

## Ethics Statement

Ethical approval for this study was received from The Ethics Committee for Social Research and Human Experimental Research Public Policy Institute Prince of Songkla University (EC001/2024).

## Conflicts of Interest

The authors declare no conflicts of interest.

## Data Availability

The data that support the findings of this study are available on request from the corresponding author. The data are not publicly available due to privacy or ethical restrictions.

## References

[1] K. Lock , “Health Impact Assessment,” BMJ (Clinical Research Ed.) 320, no. 7246 (2000): 1395–1398, 10.1136/bmj.320.7246.1395. PMC1118057 10818037

[2] Seventy‐seventh World Health Assembly , “WHA77.2 Resolution on Social Participation for Universal Health Coverage, Health and Well‐Being,” 2024, https://apps.who.int/gb/ebwha/pdf_files/WHA77/A77_R2‐en.pdf.

[3] L. Gutierrez , I. Montiel , J. A. Surroca , and J. A. Tribo , “Rainbow Wash or Rainbow Revolution? Dynamic Stakeholder Engagement for SDG‐Driven Responsible Innovation,” Journal of Business Ethics 180, no. 4 (2022): 1113–1136, 10.1007/s10551-022-05190-2. PMC9297266 35873084

[4] J. Kemm , “Health Impact Assessment: A Tool for Healthy Public Policy,” Health Promotion International 16, no. 1 (March) (2001): 79–85, 10.1093/heapro/16.1.79. 11257857

[5] IAIA , “International Association for Impact Assessment (IAIA),” 2023, https://www.iaia.org/wiki‐details.php?ID=14.

[6] J. L. Veerman , “Health Impact Assessment,” in Encyclopedia of Applied Ethics (Elsevier, 2012), 551–555, https://linkinghub.elsevier.com/retrieve/pii/B9780123739322001137.

[7] M. Wise , P. Harris , B. Harris‐Roxas , and E. Harris , “The Role of Health Impact Assessment in Promoting Population Health and Health Equity,” Health Promotion Journal of Australia 20, no. 3 (2009): 172–179. 19951236 10.1071/he09172

[8] B. Harris‐Roxas and M. O'Mullane , “Health Impact Assessment for Health Promotion, Education and Learning,” Global Health Promotion 24, no. 2 (2017): 3–4, 10.1177/1757975917704614. 28556769

[9] A. L. Dannenberg , “Effectiveness of Health Impact Assessments: A Synthesis of Data From Five Impact Evaluation Reports,” Preventing Chronic Disease 13 (2016): E84, 10.5888/pcd13.150559. 27362932 PMC4951082

[10] B. Harris‐Roxas and E. Harris , “The Impact and Effectiveness of Health Impact Assessment: A Conceptual Framework,” Environmental Impact Assessment Review 42 (2013): 51–59, 10.1016/j.eiar.2012.09.003.

[11] L. D. Broeder , E. Uiters , W. ten Have , A. Wagemakers , and A. J. Schuit , “Community Participation in Health Impact Assessment. A Scoping Review of the Literature,” Environmental Impact Assessment Review 66 (2017): 33–42, 10.1016/j.eiar.2017.06.004.

[12] T. L. Pettman , R. Armstrong , B. Pollard , et al., “Using Evidence in Health Promotion in Local Government: Contextual Realities and Opportunities,” Health Promotion Journal of Australia 24, no. 1 (2013): 72–75, 10.1071/HE12902. 23575594

[13] M. J. Morgan , E. Stratford , S. Harpur , and S. Rowbotham , “Local Government's Roles in Community Health and Wellbeing in Australia: Insights From Tasmania,” Health Promotion Journal of Australia 35, no. 4 (2023): 831, 10.1002/hpja.831. 38050655

[14] A. Carrad , I. Aguirre‐Bielschowsky , N. Rose , K. Charlton , and B. Reeve , “Food System Policy Making and Innovation at the Local Level: Exploring the Response of Australian Local Governments to Critical Food Systems Issues,” Health Promotion Journal of Australia 34, no. 2 (2023): 488–499, 10.1002/hpja.626. 35718947

[15] J. Kemm , J. Parry , and S. Palmer , “Using HIA in Local Government,” in Health Impact Assessment, ed. J. Kemm , J. Parry , and S. Palmer (Oxford University Press, 2004), 243–252, 10.1093/acprof:oso/9780198526292.003.0022.

[16] R. Mas‐Pons , M. Caturla‐Bastit , J. Bisbal‐Sanz , M. López‐Nicolás , and C. Barona‐Vilar , “Health Impact Assessment of Local Policies: Methodology and Tools,” Global Health Promotion 30, no. 1 (2022): 7–15, 10.1177/17579759221107031. 35855588

[17] M. O'Mullane and A. Quinlivan , “Health Impact Assessment (HIA) in Ireland and the Role of Local Government,” Environmental Impact Assessment Review 32, no. 1 (2012): 181–186, 10.1016/j.eiar.2011.08.004.

[18] K. Berensson and P. Tillgren , “Health Impact Assessment (HIA) of Political Proposals at the Local Level: Successful Introduction, but What Has Happened 15 Years Later?,” Global Health Promotion 24, no. 2 (2017): 43–51. 10.1177/1757975916683386 28436297

[19] L. Green , L. Parry‐Williams , and E. Huckle , Health Impact Assessment (HIA) and Local Development Plans (LDPs): A Toolkit for Practice (Public Health Wales NHS Trust, 2021), https://phwwhocc.co.uk/wp‐content/uploads/2021/10/HIA‐and‐LDPs‐Toolkit‐E‐final.pdf.

[20] C. Middleton , S. Pengkam , and A. Tivasuradej , “Politics of Knowledge and Collective Action in Health Impact Assessment in Thailand: The Experiene of the Khao Hinsorn Community,” in Water Governance and Collective Action: Multi‐Scale Challenges, ed. D. Suhardiman , A. Nicol , and E. Mapedza (Routledge/Taylor & Francis Group, 2017), 70–81.

[21] S. Pengkam , Revitalizing Thailand's Community Health Impact Assessment (National Health Commission Office, 2012), https://en.nationalhealth.or.th/wp‐content/uploads/2017/11/2012_Revitalizing‐Thais‐Community‐HIA.pdf.

[22] P. Jongudomsuk and J. Srisasalux , “A Decade of Health‐Care Decentralization in Thailand: What Lessons Can Be Drawn?,” WHO South‐East Asia Journal of Public Health 1, no. 3 (2012): 347–356, 10.4103/2224-3151.207031. 28615561

[23] Health Impact Assessment Division , Guidelines of Health Impact Assessment for Local Authority (Ministry of Public Health, 2013).

[24] Ottawa Charter for Health Promotion , “First International Conference on Health Promotion,” Ottawa, 1986. WHO/HPR/HEP/95.1, https://www.healthpromotion.org.au/images/ottawa_charter_hp.pdf.

